# Dispensers under the Insurance Act

**Published:** 1912-06-15

**Authors:** Geo. W. Udale

**Affiliations:** "Ellastone," Harlington, Middlesex


### THE EDITOR'S LETTER-BOX.

### Dispensers Under the Insurance Act.

*To the Editor of* THE HOSPITAL.

SIR,—The article on the above subject in your issue of June 8, 1912, by "A London Pharmacist," is interesting and educative to at least one of your readers, to wit myself. For the first time since this highly controversial measure was introduced, I learn, through your contributor, that the position of the dispenser under this Act is one subject to "misunderstanding and pessimism." It is, of course, a case of *Quot homines, tot sententiæ*; but, to my simple way of thinking, the position of the dispenser has never been open to question; for, in some shape or form, either with qualification or without, this position has been assured, and there has been no misunderstanding about it. As for the pessimism, this may arise through a personal feeling as to whether the dispenser would prefer the probable change from the "doctor's surgery" to the "chemist's shop."

It seems to me that if there be any doubt at all in respect of any future positions under the Act, those positions are those of the pharmacist in business for himself and the qualified assistant pharmacist.

Your contributor's forecast portends the removal of all the dispensing, etc., under the Act from the surgery to the pharmacy, with heaps of dispensers rushing after it; certainly a state of affairs highly desirable, and one that I should be delighted to see. We are still awaiting, however, the regulations of the Insurance Commissioners, and so, in the interim, I would suggest that your contributor should read, mark, learn, and inwardly digest Clause 14 of the Act; having done so, I would then refer him to your contemporary, *The Chemist and Druggist*, for January 20, 1912, folio 45, column ii., and folio 46, column i. (the same journal, in its leading article for June 8, 1912, says that "the nearer we approach the enforcement of the Act, the more nebulous the pharmaceutical position becomes," etc.); having, as a commonsense man, as all pharmacists are, or should be, thoroughly grasped those points to which I have drawn his attention, I would ask him to pen a few lines to THE HOSPITAL on the subject of "Whose position under the Insurance Act is one of misunderstanding and pessimism?"

It is certainly not that of the dispenser *per se*. With compliments,

Yours faithfully,
GEO. W. UDALE, M.P.S., Ph.C.
"Ellastone," Harlington, Middlesex, June 8.

[Our correspondent is beating the air; if, as his quotation from a contemporary suggests, the pharmaceutical position becomes more nebulous every day, there is surely ground for pessimism. The opinion of "A London Pharmacist," however, differed from that of the contemporary quoted, and our correspondent would do well to wait and see which of the two opinions is correct.—ED. THE HOSPITAL.]

